# The relationship between having a regular general practitioner (GP) and the experience of healthcare barriers: a cross-sectional study among young people in NSW, Australia, with oversampling from marginalised groups

**DOI:** 10.1186/s12875-020-01294-8

**Published:** 2020-10-28

**Authors:** Melissa Kang, Fiona Robards, Georgina Luscombe, Lena Sanci, Tim Usherwood

**Affiliations:** 1 Australian Centre for Public and Population Health Research, University of Technology Sydney, Ultimo, Australia; 2 Department of General Practice, The University of Sydney, Westmead Clinical School, Sydney, Australia; 3 School of Public Health, Room 224, Level 8, Building 10, 235 Jones St, ULTIMO, NSW 2007 Australia; 4 School of Rural Health, The University of Sydney, Orange, Australia; 5 Department of General Practice, University of Melbourne, Carlton, Australia; 6 The George Institute for Global Health, Sydney, Australia

**Keywords:** Adolescents, Health services accessibility, Health equity, General practice, Primary health care, Continuity of patient care, Survey

## Abstract

**Background:**

Young people (12–24 years) visit general practice but may not have a ‘regular’ general practitioner (GP). Whether continuity of GP care influences experiences with, and barriers to, health care among young people is unknown. This paper explores the association between having a regular GP and experience of healthcare barriers and attitudes to health system navigation among young people in New South Wales (NSW), Australia.

**Methods:**

This study was a cross-sectional survey administered either online or face-to-face in community settings. Young people living in NSW were recruited, with oversampling of those from five socio-culturally marginalised groups (those who were Aboriginal and Torres Strait Islander, homeless, of refugee background, in rural or remote locations, sexuality and/or gender diverse). In this analysis of a larger dataset, we examined associations between having a regular GP, demographic and health status variables, barriers to health care and attitudes to health system navigation, using chi-square tests and odds ratios. Content and thematic analyses were applied to free-text responses to explore young people’s views about having a regular GP.

**Results:**

One thousand four hundred and sixteen young people completed the survey between 2016 and 2017. Of these, 81.1% had seen a GP in the previous 6 months and 57.8% had a regular GP. Cost was the most frequently cited barrier (45.8%) to accessing health care generally. Those with a regular GP were less likely to cite cost and other structural barriers, feeling judged, and not knowing which service to go to. Having a regular GP was associated with having more positive attitudes to health system navigation. Free-text responses provided qualitative insights, including the importance of building a relationship with one GP.

**Conclusions:**

General practice is the appropriate setting for preventive health care and care coordination. Having a regular GP is associated with fewer barriers and more positive attitudes to health system navigation and may provide better engagement with and coordination of care. Strategies are needed to increase the proportion of young people who have a regular GP.

**Supplementary Information:**

The online version contains supplementary material available at 10.1186/s12875-020-01294-8.

## Background

Strong primary health care systems promote health equity through universal access and reduce health care costs by rationalising access to secondary care. Continuity of care and care coordination in primary health reduces rates of hospital admissions [1]. The Australian government has invested in strengthening primary care through structural reforms such as the creation of Primary Health Networks [2], review of GP reimbursements [3] and initiatives such as ‘health care homes’ for chronic complex illness [4].

It has long been recognized that general practice is the most appropriate of the medical specialties to address prevention and determinants of health [5]. In Australia, patients are not registered with a specific general practice and may choose which practice to attend on each occasion. General practice is fee-for-service, with most services eligible for rebates from the universal health insurance scheme known as Medicare. GPs choose whether to bill patients or to claim the patient’s rebate directly from Medicare. This latter practice is known as ‘bulk-billing’ and involves no upfront or ‘gap’ fee. Public hospital emergency services are free to access.

The mental health of young Australians has declined over the past decade, with 15.4% of young adults suffering high or very high psychological distress in 2014–2015 compared with 11.8% in 2011, and youth suicide rates increasing from 10.3 per 100,000 in 2007 to 12.7 per 100,000 in 2016 [6]. Suicide, followed by accidents and accidental poisoning, are the leading causes of death [7]. Additionally, while smoking, alcohol and substance use among young people has declined, overweight and obesity, and sexually transmitted infection rates continue to increase [6].

Recent data show an increase in presentations by adolescents and young adults in Australia to emergency departments, particularly for mental health problems [8, 9]. One concerning explanation for this is inadequate capacity of the health system to respond to young people’s mental health crises [10]. Australian research has described the positive health impacts of psychosocial screening among young people in general practice [11]. Barriers to help-seeking among young people include concerns about confidentiality, lack of awareness of services available, stigma, poor accessibility, perceived attitudes of health care workers, cost and inadequately trained health care providers [12]. A recent systematic review of research among marginalised young people found barriers existed across all groups but were more prominent for some [13]. Among homeless young people, cost barriers were more pronounced, while fear of discrimination appeared more salient for sexuality and gender diverse young people. For rural young people, confidentiality concerns, transport and availability of services featured more strongly. Among refugee youth, language barriers and difficulty understanding health systems presented more commonly as barriers and among Indigenous young people, culturally sensitive services were cited as particularly important [13].

It is in this context of ongoing health system reform, concerning health indicators and the need to address potential inequities of access that this study is situated. Our primary aim was to explore the barriers to health care and attitudes towards health system navigation for young Australians (12–24 years) and their association with having a regular GP. We also examined the association between having a regular GP, psychological distress and health care utilisation.

## Methods

### Design and sample

This paper reports on data from a cross-sectional survey which targeted all young people (12–24 years) residing in New South Wales (NSW), Australia, with oversampling from five socio-culturally marginalised groups: Aboriginal and/or Torres Strait Islanders, and those experiencing homelessness, of refugee background, living in rural or remote areas, or who were sexuality and/or gender diverse.

The survey was one of four discrete, interrelated studies comprising the ‘Access 3’ project which explored health system navigation for young people in the digital age, in order to inform policy. The full project protocol has been published [14].

To purposively sample marginalised young people, we worked with networks and advocates from a range of organizations in rural areas, supported accommodation services, community organisations and services who work with or for one or more of the five marginalised groups. We had two study Reference Groups (one urban, one rural) who provided survey promotion and recruitment support and advice. The survey was available online via a LimeSurvey [15] platform and in hardcopy. Survey promotion and recruitment occurred online (paid advertisements on social media, social media sharing by youth consultants and others, emails and websites) and offline (events, networks and local ‘champions’). The logistics of survey promotion and recruitment among marginalised populations of young people involved establishing governance structures with clear terms of reference and a range of ethical considerations. We have published the details of these as a case study in research methods [16].

### Measures

The survey instrument was developed with sixteen youth consultants, who provided guidance throughout the Access 3 project. One or more consultants were from each of the five marginalised groups. Questionnaire items included postcode to identify rurality using the Australian Standard Geographical Classification System [17] and expanded demographic data to capture marginalised groups, presence of chronic health conditions and/or disability, self-reported health status, psychological distress (measured via Kessler 10 [18]), health service utilization, barriers to accessing care and attitudes to health system navigation.

In the section on accessing health care, participants were asked about having a regular GP. Response options included ‘I have a regular GP/ doctor’, ‘I go to whatever GP/ doctor is available when I need to’, ‘I usually go to a hospital emergency department instead of a GP/doctor’, ‘I usually call a telephone GP/ doctor service’, ‘I don’t go to a GP/ doctor’. There was a free text box for ‘Comments’ at the end of this question.

Barriers to accessing health care were assessed using a list of 11 potential barriers and ‘none’; multiple responses could be selected. Attitudes to health system navigation were explored using a set of six statements and a 5-point Likert scale.

A copy of the full survey instrument can be found in the technical report [19] and is included as a Supplementary file.

### Analysis

The analysis presented in this paper focused on participant responses to questions about GP utilisation and having a regular GP. Quantitative analysis was conducted using SPSS version 24. Chi-square analyses examined associations between recent GP access, and having a regular GP, and a range of demographic and health status variables, barriers to health care and attitudes to health system navigation. Odds ratio calculations were performed to explore associations and effect size.

Open text responses to the ‘Comments’ box were analysed using content and thematic analysis [20]. Many of the responses were short but contained important words or phrases which could be categorised. For example: ‘whichever one bulk-bills’ (which means there is no upfront cost or gap payment for the patient, as described in the Background). In the Australian health system and in the context of this survey, ‘bulk-billing’ is understood to mean choice of GP practice is made according to absence of a cost barrier. Thus content analysis was conducted first to establish categories and enumerate their occurrence. Next the data were examined for categories within context and/ or patterns relating to having/ not having a regular GP in order to deduce broader themes.

## Results

Between February 2016 and February 2017, 1416 young people completed the survey; 1012/1416 (75.5%) did so online while 404/1416 (28.5%) completed paper surveys. The median age of the sample was 18 years (interquartile range 4 years), 68.4% were female, 28.7% male and 3.0% other gender. Eight hundred and ninety-seven (63.9%) belonged to at least one marginalised group. Of the five pre-defined groups, 169 (11.9%) were Aboriginal and/ or Torres Strait Islander, 118 (8.3%) were experiencing homelessness, 75 (5.3%) were of refugee background, 478 (33.8%) lived in rural or remote NSW and 426 (30.1%) were sexuality and/ or gender diverse.

Of the 1416 survey participants, 618 (43.6%) reported their health as very good or excellent, 729 (51.5%) had high or very high levels of psychological distress and 736 (52.0%) reported having a chronic health condition.

Most participants (1300/1416; 91.8%) had visited at least one health professional in the previous 6 months. By far the most commonly utilised professional was a general practitioner (GP), visited by 1149/1416 (81.1%) of the sample. Because general practice in Australia is a free market, Australian residents may visit any general practice regardless of location. A ‘regular GP’ implies the same GP is utilised most or all of the time regardless of health complaint, while ‘whatever GP/doctor’ implies there is no ongoing relationship with one GP, and that general practices in different locations or suburbs are utilised. Table 1 summarises participants’ utilisation of GPs.

**Table 1:** GP utilisation by young people (12–24 years) in NSW, Australia (*N* = 1416)

GP utilisation	N	%
I have a regular GP/doctor	819	58.3
I go to whatever GP/doctor is available when I need to	511	36.3
I usually go to a hospital emergency department instead of a GP/doctor	14	1.0
I usually call a telephone GP/doctor service	9	0.6
I don’t go to a GP/doctor	53	3.8
No response	10	0.7
**Total**	**1416**	**100.0**

Adolescents (12–17 years) were more likely than young adults (18–24 years) to have a regular GP. There was no association with gender. Among each of the marginalised groups, homeless participants were less likely to have a regular GP than those who were not homeless. Having a chronic condition was associated with having a regular GP, whether or not the participant also belonged to one or more of the five marginalised groups. (Table 2).

**Table 2:** Associations between having a regular GP and age group, gender, marginalised group or having a chronic health condition

	N	Has a regular GP n (%)	Does not have a regular GP n (%)	OR [95% CI]	***P*** value
Adolescents (12 – 17 yrs)	694	427 (61.5)	267 (38.5)	ref	**0.014**
Young adults (18–24 years)	712	392 (55.1)	320 (44.9)	0.77 [0.62–0.95]	
Female	961	549 (57.1)	412 (42.9)	ref	0.40
Male	403	243 (60.3)	160 (39.7)	1.14 [0.90–1.44]	
Other gender	42	27 (64.3)	15 (35.7)	1.35 [0.71–2.57]	
Aboriginal and/or Torres Strait Islander	164	88 (53.7)	76 (46.3)	0.81 [0.58–1.12]	0.20
Homeless	118	56 (47.5)	61 (52.5)	0.62 [0.43–0.91]	**0.015**
Refugee	71	44 (62.0)	27 (38.0)	1.18 [0.72–1.93]	0.50
Rural/Remote	471	281 (59.7)	190 (40.3)	1.08 [0.86–1.35]	0.51
Sexuality and/or Gender diverse	425	251 (59.1)	174 (40.9)	1.05 [0.84–1.33]	0.66
Has at least one chronic condition	734	456 (62.1)	278 (37.9)	ref	**0.002**
No chronic conditions	672	363 (54.0)	309 (46.0)	1.40 [1.13–1.73]	
Has at least one chronic condition, does not belong to any of the marginalised groups	239	153 (64.0)	86 (36.0)	ref	**0.047**
No chronic conditions, regardless of whether marginalised or not	1167	666 (57.1)	501 (42.9)	1.34 [1.003–1.79]	

The proportion of participants who had a recent (prior 6 months) consultation with a GP increased linearly with increasing levels of psychological distress, from 77.6% (274/353) of those with low distress to 85.0% (346/407) of those with very high distress, *p* = 0.005. However there was no relationship between psychological distress and whether the respondent had a regular GP. Of those with high or very high K10 scores, 58.2% (428/727) reported having a regular GP, similar to the 58.0% (386/666) of those with low or moderate K10 scores.

Participants could select one or more barriers to health care, being defined as [something] ‘that would stop or prevent you from going to a health service’. Cost was the most frequently cited, by 45.8% (649/1416) of the sample.

Participants who had a regular GP were less likely to cite cost, feeling judged, not knowing which service to go to, opening hours and ‘not having their own Medicare card’ (see Table 3 footnote for explanation). Further, those with a regular GP were more likely to report ‘no barriers’ compared to those without a regular GP. See Table 3.

**Table 3:** Association between having a regular GP and barriers to access (*n* = 1406)

	Has a regular GP n (%)	Does not have a regular GP n (%)	***P*** value
Cost	350 (42.7)	298 (50.8)	**0.003**
Opening hours mean I need time off work or study	231 (28.2)	218 (37.1)	**< 0.0001**
I would feel embarrassed	215 (26.3)	176 (30.0)	0.12
Difficulty getting there	181 (22.1)	154 (26.2)	0.07
I would have to ask my parents/carers to take me	189 (23.1)	124 (21.1)	0.39
I would feel judged	152 (18.6)	135 (23.0)	**0.04**
The gender of the doctor/ health professional	158 (19.3)	108 (18.4)	0.67
I worry about confidentiality	118 (14.4)	99 (16.9)	0.21
I don’t have my own Medicare card^a^	88 (10.7)	85 (14.5)	**0.04**
I don’t know which service to get to	81 (9.9)	84 (14.3)	**0.01**
Language or cultural reasons	46 (5.6)	35 (6.0)	0.78
No barriers	191 (23.3)	108 (18.4)	**0.03**

^a^A Medicare card is physical evidence of Medicare eligibility even though presenting the card is not essential for receiving a service. Young people can apply for their own card from 15 years of age; those who remain on their family’s Medicare card will not physically own a card but can still seek confidential health care

Attitudes towards navigating the health system were generally positive, with almost three-quarters of the sample (1037/1416) agreeing that they could find and access appropriate health services when they needed them, and 64.3% (911/1416) agreeing that they have a good understanding of the different health services available to them. Having a regular GP was associated with having a better understanding of the different available health services, having experience using multiple different services and with being less inclined to prefer online over face to face consultation. See Table 4.

**Table 4:** Association between having a regular GP and attitudes towards navigating the health system (*n* = 1394)

Strongly Agree/ Agree	I have a regular GP n (%)	No regular GP n (%)	***P*** value
I get confused by the number of different health services available	230 (28.4)	161 (27.5)	0.71
I have a good understanding of the different health services that are available to me	551 (68.1)	357 (61.0)	**0.01**
I can find and access appropriate health services when I need them	612 (75.7)	422 (72.1)	0.13
I have had to visit too many different services unnecessarily	143 (17.7)	94 (16.1)	0.44
I have been to lots of different services because I needed to	363 (45.0)	172 (29.5)	**< 0.0001**
I would prefer to access online services than physically go to a health service for some health issues but not others	216 (26.8)	221 (37.8)	**< 0.0001**

One hundred and eighty-three participants provided open text responses at the end of the question about GP utilisation. Of these, 90 had selected that they had a regular GP, 80 that they go to whatever GP is available when they need one, 11 that they don’t go to the GP and two that they go to the emergency department instead of a GP.

Content analysis provided useful information about the 170/183 who provided open text responses and had selected that they had a regular GP or that they went to whatever GP is available. Eighteen of the 90 who had a regular GP commented that they sometimes had to see a different GP at the same practice due to the unavailability or popularity of their regular GP which led to unacceptable waiting times for an appointment. A further 12 described visiting different practices if their regular GP was unavailable.

Of the 80 participants who attended ‘whatever GP is available when they need one’, 20 did in fact always attend the same practice, and sometimes indicated that they also had a preferred GP there and a further 12 had ‘preferred GPs’ but would attend different practices based on convenience or cost. Seven described cost and convenience as the reasons for attending ‘whatever GP is available when needed’. Fifteen of these 80 participants described looking for and wanting a regular GP.

In summary, there were some similarities and overlap in the way participants with and without a regular GP actually utilised general practice. One third of those with regular GPs did sometimes visit other GPs in the same, or a different practice. One quarter of those who went to ‘whatever GP is available’ did attend a regular practice.

Thematic analysis involved a more nuanced and contextual examination of the data. Three themes emerged:

1. The value of a regular GP

Participants with a regular GP described factors such as trust, feeling comfortable and efficiency as reasons why having a regular GP was important to them. Several described having complex health issues and wanting consistency and continuity of care.

(2) The desire to have a regular GP

Wanting and looking for a regular GP was a prominent theme among participants who did not have one. Some described structural barriers to being able to find one (such as cost, location and availability of GP) while others expressed the difficulty in finding a GP they felt comfortable with

(3) Split care between a regular GP and other GPs, or between non-regular general practices.

Split care describes the deliberate utilisation of two or more general practices and was another prominent theme. This occurred among participants with and without a regular GP, for reasons such as convenience, cost and location.

Illustrative quotes for each of the three themes are provided in Table 5.

**Table 5:** Participants’ perspectives on having a regular GP. NB each of the thirteen quotes represents 13 different participants

**The value of having a regular GP**	
*After some bad/judgemental experiences with random doctors I learnt the importance of travelling/making an effort to develop a relationship with one GP* (Female, 22 years)	
*I have developed a close trust with my GP, and given that she knows my past history, it is much easier to see her about any health related problems.* (Female, 22 years)	
*I used to go to whatever GP was available but I find a consistent record of my medical history helps move things along easier.* (Male, 22 years)	
**Split care is common due to structural barriers despite a preference for a regular GP**	
*My preferred doctor is very popular, and to get in you need to make an appointment months ahead, so when I am sick I need to go to a different one with more availability* (Female, 17 years)	
*I prefer to have a regular GP but if they’re not available I’ll go to anyone* (Female, 20 years)	
*I used to have a regular GP, however the cost was too much when I was having to regularly see them for things such as renewing prescriptions or getting a regular injection* (Female, 19 years)	
*Two doctors - one that bulk bills the other ($70 per appointment) only if multiple issues.* (Female, 20 years)	
*I like to have multiple doctors to visit as one it is more convenient if one is away and also different doctors give different advice. For example I find one of my doctors more drug orientated to solve my health issues whereas another doctor takes more of a lifestyle change approach. I once had a suspected iron deficiency and one doctor told me to write down everything I ate for a week and told me to visit her in a week and another ordered a blood test. The second was much more convenient as we live in busy lifestyles and demand quick fix solutions* (Female, 16 years)	
*When at home I have a family GP when at uni I go to uni health service & whichever doctor is available* (Female, 19 years)	
**A regular practice is a good compromise or the only practical solution**	
*I attend a regular practice, however see any doctor that is available when I go.* (Female, 22 years)	
*It changes a bit but usually I try to stick with the same practice so most of my history and such is there* (Female, 19 years)	
*I don’t necessarily have a regular doctor but go to the same clinic usually.* (Female, 21 years)	
*I attend my local medical centre, I try to see the same doctor each time however there is one other doctor I will see if he is not there. Only when I urgently need to see a doctor I will see first available, otherwise I will wait for a few days.* (Female, 18 years)	

## Discussion

Our study, consisting of 1416 young people aged 12 to 24 years, is unique in its inclusion of those from a range of marginalised groups. Cost was the most prominent barrier to health care access for each of the five marginalised groups, for those with a chronic illness, and for those who did not belong to any of these subsamples. Young people with a regular GP were less likely to report cost and several other barriers, and to have more positive attitudes to health system navigation.

The finding that cost was the most frequently reported barrier for young people is new. Increasing out-of-pocket costs of health care have been implicated in the rise in emergency presentations [21] and foregone care [22].

The proportion of young people in our study who had a regular GP was substantially less than in the general population. The Royal Australian College of General Practitioners recently reported that 78.2% of patients have a preferred GP and that those with a usual GP report better experiences of care [23]. As a free-market system, general practice in Australia allows people to visit any GP or practice. It is possible that older people have had more experience and time to establish a relationship with one GP or practice.

We found that having a regular GP was associated with lower reporting of structural barriers such as cost, opening hours and not owning a Medicare card, and was less likely to be associated with feeling judged. This may reflect better engagement, trust, and familiarity with the health system, as well as care coordination and continuity of care. Another reason for reduced cost barriers could be that regular GPs reduce their own fees or choose bulk-billing for selected patients [24] or advocate for their young clients when making referrals. A recent study with GPs in a socioeconomically disadvantaged area of metropolitan Sydney, Australia found that GPs spent time trying to find affordable options for their patients when referring them to specialists [25]. This area also has a high proportion of refugees and Aboriginal people. A narrative synthesis looking at primary health care delivery models for refugees in resettlement countries identified that in Australia and the US, many models of care involved negotiation of pro bono medical services [26]. In relation to young people, the NSW Health Department has published a clinical resource for GPs [27] which is endorsed by the Royal Australian College of General Practitioners and which is included in NSW GP training programs. This training includes advocacy as an important role that GPs can play in supporting young people to access health care by reducing barriers such as cost.

The associations between continuity of care and outcomes such as reduced hospitalisations, improved adherence to medication, and lower mortality have been demonstrated in studies involving older people or those with chronic diseases [28, 29]. Our study suggests that continuity of care could be just as important for young people, particularly those who are marginalised or have chronic health conditions, including mental health problems.

It is reasonable to assume that efforts to increase the proportion of young people who have a regular GP are warranted. GPs need adequate time and focussed effort to engage with young people, but this is difficult in a fee-for-service funding model. A related Access 3 study among health professionals found that GPs could reinforce fragmentation of care through referral patterns, which tend to be to private specialists. GPs might be less aware of free or low cost public services and many health professionals underestimate the impact of cost on young people’s ability to navigate the health system [30]. A ‘regular GP’ could provide navigation support by considering cost and other barriers, assertively following up referrals and advocating for young patients when necessary.

The limitations of this study include its cross-sectional design. Associations between variables do not imply causality, however the inclusion of qualitative data strengthen the quantitative findings that having a regular GP has benefits for and is valued by young people. It is possible that young people with a regular GP were more health literate and well-versed in navigating the health system. We used a range of recruitment strategies that were not uniform, guided by stakeholders (including young people, professionals and advocates). While this approach introduced sampling bias, it did help us recruit participants who might not otherwise have been reached and provided valuable lessons for future research and research translation involving marginalised young people. Further, to our knowledge this is one of the first studies internationally to include and oversample from multiple marginalised groups of young people and gave us sufficient power to make some comparisons between groups. Our sample was self-selected which also impacts on representativeness. In particular less than one third of our sample (28%) were males. Nevertheless, the perspectives of marginalised and often chronically unwell young people may provide a more critical look at health system challenges.

Our findings reinforce that GPs remain the most frequently utilised primary health care provider in the Australian health system [31], even for young people who are marginalised, as well as those with chronic health conditions who require clinical care from a range of primary and secondary health services. Our qualitative analyses revealed a pattern of utilisation of more than one GP or practice and deliberate ‘splitting of care’ even for young people who had, or desired, a regular GP. This is consistent with other research in Australia that has found that over one-quarter of adults attend multiple general practices even when they have a usual practice [32] and that decisions around utilising multiple practices take into consideration waiting times, preference for specific GPs and the reason for needing to see a GP [33].

Our study has implications for general practice and primary care. If having a regular GP is preferred by young people and improves their engagement and access, there need to be policy and practice mechanisms for facilitating this. In 2015, the Royal Australian College of General Practitioners recommended the introduction of voluntary patient enrolments to enhance continuity of care [34]. This was to have been introduced in 2020 by the Australian Government initially for people aged 70 years and older (or 50 years and older for Aboriginal and Torres Strait Islander people) who have chronic and complex conditions, but has been delayed due to the Covid19 pandemic. Advocating for marginalised young people with complex chronic health conditions to be included and evaluate a range of outcomes will be an important way to seek equity. Ensuring that GPs are well trained in skills for engaging young people is also critical. The existing fee structures seem to be inadequate for providing comprehensive assessments and time for building therapeutic relationships. GPs could invite young patients to return to them for follow-up and for their ongoing care needs so that they have continuity of care.

Alternative solutions include different models of care that are less reliant on fee-for-service general practice. In Victoria, a ‘Doctors in Schools’ program is being rolled out among 100 disadvantaged schools, and implemented via Primary Health Networks [35]. Alternate settings where young people can be easily reached could be considered, as well as digital pathways to supplement face to face care.

Primary care professionals other than GPs could complement the work of GPs. Pilot nurse-led youth clinics in general practice in rural Victoria were not feasible in the short term but provided valuable lessons for future programs [36]. Health care navigators are an evolving role in health systems, often fulfilled by nurses, and particularly target disadvantaged or vulnerable populations or those with chronic and complex illness [37].

There is an opportunity to improve young people’s health literacy about health system navigation. For example, in school young people could learn about why having a ‘regular GP’ is important alongside other information to increase understanding of the health system and its navigation.

To achieve equity in health and universal health coverage, hallmarks of strong primary health care, ongoing efforts are needed to support general practice as well as young people to build trusting and ongoing relationships and coordination and continuity of care.

## Conclusions

Having a regular GP is associated with fewer barriers and more positive attitudes to health system navigation and might facilitate better engagement with, and continuity and coordination of care among, adolescents and young adults. Strategies are needed to increase the proportion of young people who have a regular GP.

## Supplementary Information

**Additional file 1: Appendix 1**. NSW Youth Health Survey

## Data Availability

The data that support the findings of this study are available on request from the corresponding author [MK]. The data are not publicly available due to them containing information that could compromise research participant privacy/consent.

## Abbreviations

GP: General Practitioner
K10: Kessler-10
NSW: New South Wales
SPSS: Statistical Package for the Social Sciences

## References

[1] Hoffmann K, Stein KV, Maier M (2013). Access points to the different levels of health care and demographic predictors in a country without a gatekeeping system. Results of a cross-sectional study from Austria. Eur J Pub Health.

[2] Australian Government Department of Health (2018). Fact Sheet: Primary Health Networks.

[3] Australian Government Department of Health 2019. Australia’s long term national health plan. https://wwwhealthgovau/sites/default/files/australia-s-long-term-national-health-plan_0pdf Accessed 29 May 2020.

[4] Australian Institute of Health and Welfare (2018). Australia’s health 2018. Australia’s health series no. 16. AUS 221.

[5] Roberts J, Sanci L, Haller D (2012). Global adolescent health: is there a role for general practice?. Br J Gen Pract.

[6] Australian Research Alliance for Children and Youth (ARACY). Report Card 2018: the wellbeing of young Australians: ARACY; 2018. https://www.aracy.org.au/publications-resources/command/download_file/id/361/filename/ARACY_Report_Card_2018.pdf . Accessed 3 June 2020.

[7] Australian Institute of Health and Welfare. Deaths in Australia: web report: Cat no: PHE 229; 2018. https://wwwaihwgovau/reports/phe/229/deaths/deaths-in-australia/contents/life-expectancy Accessed 29 May 2020.

[8] Hiscock H, Neely RJ, Lei S (2018). Paediatric mental and physical health presentations to emergency departments, Victoria, 2008e15. Med J Aust.

[9] Perera J, Wand T, Bein KJ (2018). Presentations to NSW emergency departments with self-harm, suicidal ideation, or intentional poisoning, 2010e2014. Med J Aust.

[10] Sawyer SM, Patton GC (2018). Why are so many more adolescents presenting to our emergency departments with mental health problems? [Editorial]. Med J Aust.

[11] Sanci L, Chondros P, Sawyer S (2015). Responding to young people’s health risks in primary care: a cluster randomised trial of training clinicians in screening and motivational interviewing. PLoS One.

[12] Anderson JE, Lowen CA (2010). Connecting youth with health services: systematic review. Can Fam Physician.

[13] Robards F, Kang M, Sanci LA (2018). How marginalised young people access, engage with and navigate healthcare systems in the digital age: systematic review. J Adolesc Health.

[14] Kang M, Robards F, Sanci L (2017). Access 3 project protocol: young people and health system navigation in the digital age: a multifaceted, mixed methods study. BMJ Open.

[15] LimeSurvey GH (2003). LimeSurvey: an open source survey tool /LimeSurvey GmbH, Hamburg, Germany.

[16] Robards FJ, Kang M, Sanci L, et al. Methods research: exploring health system access, engagement, and navigation, SAGE research methods cases: medicine and health, 2020. 10.4135/9781529741520 ISBN: 9781529741520.

[17] Australian Bureau of Statistics (ABS). 1270.0.55.005 - Australian Statistical Geography Standard (ASGS): Volume 5 – Remoteness Structure, July 2016: ABS; 2018. https://wwwabsgovau/ausstats/abs@nsf/Latestproducts/1270055005Main%20Features15July%202016?opendocument&tabname=Summary&prodno=1270055005&issue=July%202016&num=&view= Accessed 8 October 2020.

[18] Kessler RC, Andrews G, Colpe LJ, Hiripi E, Mroczek DK, Normand SLT (2002). Short screening scales to monitor population prevalences and trends in non-specific psychological distress. Psychol Med.

[19] Kang M, Robards F, Sanci L, Steinbeck K, Jan S, Hawke C, Luscombe G, Kong M, Usherwood T. *Access 3*: young people and the health system in the digital age - final research report. In: Department of General Practice Westmead, the University of Sydney and the Australian Centre for Public and Population Health Research, the University of Technology Sydney, Australia: ISBN; 2018. 978-1-74210-455-3.

[20] Marks DF, Yardley L (2004). Content and thematic analysis, in: research methods for clinical and Health Psychology, London SAGE Publications.

[21] Authority NHP (2016). Healthy Communities: Use of emergency department and GP services in 2013–14.

[22] Laba T, Usherwood T, Leeder S (2014). Co-payments for health care: what is their real cost?. Aust Health Rev.

[23] Royal Australian College of General Practitioners (2018). General practice: health of the nation 2018.

[24] Beaton A, Usherwood T, Leeder SR, Russell LM. Perceptions of economic hardship and implications for illness management: a survey of general practitioners in western Sydney: Menzies Centre for Health Policy; 2011. ISBN 978-0-9807442-5-5.

[25] Reath J, King M, Kmet W, O’Halloran D, Brooker R, Aspinall D, Bittar H, Seelan T, Burke M, Usherwood T. Experiences of primary healthcare professionals and patients from an area of urban disadvantage: a qualitative study. BJGP Open. 2019;3(4) bjgpopen19X101676. DOI. 10.3399/bjgpopen19X101676. 10.3399/bjgpopen19X101676 PMC6995866 31772038

[26] Joshi C, Russell G, Cheng I (2013). A narrative synthesis of the impact of primary health care delivery models for refugees in resettlement countries on access, quality and coordination. Int J Equity Health.

[27] Chown P, Kang M, Sanci L, Newnham V, Bennett DL. Adolescent health: enhancing the skills of general practitioners in caring for young people from culturally diverse backgrounds, GP resource kit. 2nd ed: NSW Centre for the Advancement of Adolescent Health and Transcultural Mental Health Centre, Sydney; 2008. ISBN: 9780980495102.

[28] Pereira Gray DJ, Sidaway-Lee K, White E (2018). Continuity of care with doctors—a matter of life and death? A systematic review of continuity of care and mortality. BMJ Open.

[29] Jeffers H, Baker M. Continuity of care: still important in modern-day general practice. Br J Gen Pract. 2016:396–7. 10.3399/bjgp16X686185. 10.3399/bjgp16X686185 PMC4979920 27481958

[30] Robards F, Kang M, Tolley K, et al. Marginalised young people’s healthcare journeys: professionals' perspectives. Health Educ J. 2018. 10.1177/0017896917752965.

[31] Australian Bureau of Statistics. Patient experiences in Australia: summary of findings. 2018–2019. https://www.abs.gov.au/statistics/health/health-services/patient-experiences-australia-summary-findings/latest-release#general-practitioners. Accessed 8 October 2020.

[32] Wright M, Hall J, van Gool K (2018). How common is multiple general practice attendance in Australia?. AJGP.

[33] Cosgriff D, Reath J, Abbott P. Why do people with long term health needs see more than one GP?: a qualitative study. Australian Journal of Primary Health*, in press*. 10.1071/PY20179 33292926

[34] Royal Australian College of General Practitioners (RACGP) (2015). Vision for general practice and a sustainable healthcare system.

[35] Victoria State Government Education and Training. Doctors in Secondary Schools. Updated 27 November 2019. https://www.education.vic.gov.au/about/programs/Pages/doctors.aspx?Redirect=1 Accessed 3 June 2020.

[36] Hegarty K, Parker R, Newton D (2013). Feasibility and acceptability of nurse-led youth clinics in Australian general practice. Aust J Prim Health.

[37] McMurray A, Cooper H (2017). The nurse navigator: an evolving model of care. Collegian.

