# Supplementary material for: The relationship between having a regular general practitioner (GP) and the experience of healthcare barriers: a cross-sectional study among young people in NSW, Australia, with oversampling from marginalised groups
Source: BMC Fam Pract. 2020 Oct 28;21:220. doi: 10.1186/s12875-020-01294-8 (PMC7592545; doi:10.1186/s12875-020-01294-8)
Supplement: Supplementary file 1 — Additional file 1: Appendix 1. NSW Youth Health Survey [file 12875_2020_1294_MOESM1_ESM.pdf]

# Appendix 1 – NSW Youth Health Survey

## This survey may be copied and used with or without modification provided the source is acknowledged:

Kang M, Robards F, Sanci L, Steinbeck K, Jan S, Hawke C, Luscombe G, Kong M, Usherwood T. (2018). *Access 3*: young people and the health system in the digital age - final research report. Department of General Practice Westmead, The University of Sydney and the Australian Centre for Public and Population Health Research, The University of Technology Sydney, Australia.

Please note formatting and images used in the online and paper surveys for the *Access 3* study have been removed.

# NSW YOUTH HEALTH ACCESS SURVEY

**All * indicate mandatory questions**

**Before we start, please tell us how you found out about the survey?**
**(Please tick as many as apply)***

☐ A friend
☐ Facebook
☐ Twitter
☐ Instagram
☐ Email
☐ A teacher/ lecturer/ employer/ colleague from TAFE, Uni, work mentioned it
☐ Parent/ carer
☐ Youth worker
☐ A health professional
☐ Other (please specify):

| PART 1: YOU AND YOUR INTERNET USE | |
|---|---|
| 1. How old are you? * (Please tick one) | ☐ 12 years<br>☐ 13 years<br>☐ 14 years<br>☐ 15 years<br>☐ 16 years<br>☐ 17 years<br>☐ 18 years<br>☐ 19 years<br>☐ 20 years<br>☐ 21 years<br>☐ 22 years<br>☐ 23 years<br>☐ 24 years |
| 2. What is the postcode where you live? * | |
| 3. What is the name of your suburb or your nearest town? * | |
| 4. What country were you born in? * (Please tick one) | ☐ Australia<br>☐ Other (please specify): |
| 5. What language do you mainly speak at home? * (Please tick one) | ☐ English<br>☐ Other (please specify): |
| 6. Do you have access to the internet? * (Please tick one) | ☐ Yes<br>☐ No<br>☐ Other (please specify): |
| 7. Do you have your own mobile phone? * (Please tick one) | ☐ Yes, a mobile phone (without internet access)<br>☐ Yes, a smart phone (with internet access)<br>☐ No |
| 8. How much time do you spend online? * (Please tick one) | ☐ I don't spend time online<br>☐ Every couple of days, not every day<br>☐ Less than 2 hours per day<br>☐ 2-6 hours per day<br>☐ 6-10 hours per day<br>☐ More than 10 hours per day |

9. When are you most active online? * (Please tick one)

- ☐ Early morning (5am - 9am)
- ☐ Mid-morning (9am - 12noon)
- ☐ Early afternoon (12noon - 3pm)
- ☐ Mid-afternoon (3pm - 6pm)
- ☐ Evening (6pm - 11pm)
- ☐ Late night (11pm - 5am)

## PART 2: MORE ABOUT YOU

## ALL YOUR ANSWERS ARE CONFIDENTIAL

10 Are you: * (Please tick one)

- ☐ Female
- ☐ Male
- ☐ Other (please describe):

Please feel free to tell us more about your gender/ gender identity if you want to:

11. Are you Aboriginal and/ or Torres Strait Islander? *(Please tick one)

- ☐ No, I am neither
- ☐ Yes, Aboriginal
- ☐ Yes, Torres Strait Islander
- ☐ Yes, both Aboriginal and Torres Strait Islander
- ☐ I'm not sure

12. If born overseas, did you or your family move to Australia as a refugee or asylum seeker? *(Please tick one)

- ☐ Yes
- ☐ No
- ☐ I'm not sure(please comment):
- ☐ Not applicable – I was born in Australia

13. What is your religion? *(Please tick one)

- ☐ No religion
- ☐ I'm not sure
- ☐ Christian
- ☐ Muslim
- ☐ Jewish
- ☐ Buddhist
- ☐ Hindu
- ☐ Other religion (please describe):

14.Do you have an intersex variation? (Please tick one)

(*Intersex* is when someone is born with physical sex characteristics that are not typical. There are many different variations.)

- ☐ No
- ☐ Yes
- ☐ I don't know
- ☐ I'd rather not say

15. What is your current living situation? *(Please tick one)

- ☐ I live in my family home with both parents/carers
- ☐ I live in my family home with one parent/carer
- ☐ I move between two family homes because my parents/carers do not live together
- ☐ I live with other relatives
- ☐ I live in foster care
- ☐ I live with my partner
- ☐ I live in a share house/ flat with other people
- ☐ I live in boarding school
- ☐ I live on campus at uni
- ☐ I live with a friend's family
- ☐ I live by myself
- ☐ I live in a refuge/supported accommodation
- ☐ I stay with friends/couch surf in different homes
- ☐ I live in a boarding house
- ☐ I sleep on the street/outside
- ☐ Other (please describe):

| PART 3: MORE ABOUT YOU | |
|---|---|
| 16. Are you: * (Please tick one) | □ In high school<br>□ In an Intensive English Centre (IEC) in high school<br>□ In full time university or TAFE<br>□ In part time university or TAFE<br>□ Doing other studies (please describe)<br>□ Not studying at all |
| 17. Are you: * (Please tick one) | □ In full time paid work<br>□ In part time or casual work<br>□ A carer or doing home duties full time or part time<br>□ Unemployed: looking for work **(Go to Q20)**<br>□ Unemployed: not looking for work **(Go to Q20)**<br>□ Unable to work due to sickness or disability **(Go to Q20)**<br>□ Other (please describe) |
| 18. How many paid hours do you work each week? * | |
| 19. What is your average weekly income from work (before tax)? * (Please tick one) | □ $1-$49;<br>□ $50-$99;<br>□ $100-$199<br>□ $200-$399<br>□ $400-$599<br>□ More than $600 |
| 20. Do you receive any youth allowance from the government? *(Please tick one) | □ Yes<br>□ No<br>□ I'm not sure |
| 21. Do you have your own Medicare card that just belongs to you? * (Please tick one)<br><br>(This is the green coloured card that the Government gives to families or individuals aged 15 and over to access health care) | □ Yes<br>□ No<br>□ I'm not sure |
| 22. Do you have a health care card? *<br><br>(A health care card is a type of concession card for health care that you may have received from Centrelink – it allows you to access medicine at a cheaper rate as well as some health services.) | □ Yes<br>□ No<br>□ I'm not sure |
| 23. Are you covered by private health insurance? * (e.g. Medibank Private, HCF, BUPA) (Please tick one) | □ Yes<br>□ No<br>□ I'm not sure |
| **These questions ask about sexual identity and sexual attraction.**<br>Many young people are still finding out about who they are, which is OK. | |
| 24. Which of these statements do you most agree with? *(Please tick one) | □ I am attracted only to people of a different sex/ gender<br>□ I am attracted only to people of my own sex/ gender<br>□ I am attracted to people of more than one sex/ gender<br>□ I am not sure who I'm attracted to<br>□ I don't feel attracted to people of any sex/ gender<br>□ I'd rather not say |
| 25. Which of these statements do you most agree with? *(Please tick one) | □ I am heterosexual (straight)<br>□ I am gay<br>□ I am lesbian<br>□ I am bisexual<br>□ I am queer<br>□ I am questioning my sexual identity<br>□ I'm not sure/ don't know<br>□ I am asexual/ not sexual<br>□ I'd rather not say<br>□ Other (please describe): |

## PART 4: ACCESSING HEALTH CARE

In this section, **'visit a health service'** means a place you physically go to for health care (e.g. the doctor, dentist, hospital, counsellor at school or an office, chemist etc.).

By **'online services'** we mean websites, apps or social media sites where you can interact with a health professional or a website program

**Please indicate how much you agree with each of the following statements:**

26. I use the internet to help me work out if **I need to** visit a health service. *
(Please tick one)
- ☐ Frequently
- ☐ Sometimes
- ☐ Not at all

27. I use the internet to decide **which** health service(s) I will go to. *
(Please tick one)
- ☐ Frequently
- ☐ Sometimes
- ☐ Not at all

28. I believe information on the internet **is as good as** visiting a doctor or health service for my health concerns. *
(Please tick one)
- ☐ Strongly agree
- ☐ Agree
- ☐ Not sure
- ☐ Disagree
- ☐ Strongly disagree

29. How do you decide where to go? For example, do you use the internet to help you find information about health services you can go to, or do you ask other people?
(Please describe):

30. How does the internet help you decide about whether to go to a health service?
(Please describe):

**31. Please tick one box for each of the following six statements:** *

| | Strongly agree | Agree | Not sure | Disagree | Strongly disagree |
|---|---|---|---|---|---|
| **I get confused by the number of different health services available** | | | | | |
| **I have a good understanding of the different health services that are available to me** | | | | | |
| **I can find and access appropriate health services when I need them** | | | | | |
| **I have had to visit too many different services unnecessarily** | | | | | |
| **I have been to lots of different services because I needed to** | | | | | |
| **I would prefer to access online services than physically go to a health service for some health issues but not others.** | | | | | |

32. For which health issues would you prefer to access online services?

33. For which health issues would you prefer to visit a health service?

## PART 5: ACCESSING HEALTH CARE CONTINUED

34. Which best describes you? *
(Please tick one)

- ☐ I have a regular GP/doctor
- ☐ I go to whatever GP/doctor is available when I need to
- ☐ I usually go to a hospital emergency department instead of a GP/doctor
- ☐ I usually call a telephone GP/doctor service
- ☐ I don't go to a GP/doctor

Comments:

35. Would any of the following prevent/stop you from going to a health service? *
(Tick as many as apply)

- ☐ I worry about confidentiality
- ☐ Cost
- ☐ I don't have my own Medicare card
- ☐ I would feel embarrassed
- ☐ I would feel judged
- ☐ Difficulty getting there
- ☐ Opening hours mean I need time off study or work
- ☐ I would have to ask my parents/ carers to take me
- ☐ I don't know which service/s to go to
- ☐ The gender of the doctor/ health professional
- ☐ Language or cultural reasons
- ☐ None

36. Are there any **other** reasons that you would find it hard to access health services?

37. What would make it easier for you to access health services?

38. Do you have any problems using online health services? *
(Please tick one)
Please comment:

- ☐ Yes
- ☐ No
- ☐ I'm not sure

39. What would make it easy or easier for you to access online health services?

## PART 6: YOUR EXPERIENCE ACCESSING HEALTH SERVICES (INCLUDING ONLINE SERVICES) IN THE PAST 6 MONTHS

The next questions are about **your experiences** of accessing health care in the **past 6 months:**

40. **In the past 6 months** have you used the internet to find information about how to keep yourself healthy? *
(Please tick one)
Please comment:

- ☐ Yes
- ☐ No **(Go to Q43)**

41. Did you find what you were looking for? *
(Please tick one)

Please comment:

- ☐ Yes
- ☐ No
- ☐ Not sure

| | |
|---|---|
| 42. Could you trust the information you found? * (Please tick one)<br><br>Please comment: | ☐ Yes<br>☐ No<br>☐ Not sure |
| 43. **In the past 6 months** have you used the internet to get information about health problems you have experienced? *<br>(Please tick one)<br><br>Please comment: | ☐ Yes<br>☐ No **(Go to Q46)** |
| 44. Did you find what you were looking for? *<br>(Please tick one)<br><br>Please comment: | ☐ Yes<br>☐ No<br>☐ Not sure |
| 45. Could you trust the information you found? * (Please tick one)<br><br>Please comment: | ☐ Yes<br>☐ No<br>☐ Not sure |
| 46. **In the past 6 months** have you used the internet to get information about how to visit a health service? *<br>(Please tick one)<br><br>Please comment: | ☐ Yes<br>☐ No **(Go to Q48)** |
| 47. Did you find what you were looking for? *<br>(Please tick one)<br><br>Please comment: | ☐ Yes<br>☐ No<br>☐ Not sure |
| 48. **In the past 6 months** have you used any internet based programs or apps so that you could manage health issues yourself? *<br>(Please tick one)<br><br>Please comment: | ☐ Yes<br>☐ No **(Go to Q51)** |
| 49. Were they helpful? *<br>(Please tick one)<br><br>Please comment: | ☐ Yes<br>☐ No<br>☐ Not sure |
| 50. Would you recommend them to other young people? *(Please tick one)<br><br>Please comment: | ☐ Yes<br>☐ No<br>☐ Not sure |

| | |
|---|---|
| 51. **In the past 6 months** have you been to any of the following * <br>(Please tick all that apply) | ☐ GP/doctor<br>☐ Counsellor/psychologist<br>☐ Dentist<br>☐ Medical specialist (e.g. a skin specialist, surgeon)<br>☐ Nutritionist/dietitian/food expert<br>☐ Nurse<br>☐ Psychiatrist (Doctor specialising in mental health)<br>☐ Paediatrician<br>☐ Physiotherapist<br>☐ Pharmacist/chemist<br>☐ Traditional healer (please specify):<br>☐ None<br>☐ Other health professional (please specify): |
| 52. **In the past 6 months,** have you been to any of the following? * <br>(Please tick all that apply) | ☐ Online service where you interact with people (e.g. eheadspace)<br>☐ headspace centre<br>☐ Youth health service<br>☐ Aboriginal Medical Service<br>☐ Family planning service<br>☐ Sexual health clinic<br>☐ Mental health service<br>☐ Drug and alcohol service<br>☐ School counsellor<br>☐ Admitted to hospital<br>☐ Emergency Department<br>☐ None<br>Other (please describe): |

*If you answered "none" to **Q51** **and** **Q52** please skip to Q59.

53. Can you tell us in a few words why you went to this/ these health service/s?

54. How did you decide where to go?

55. How did you use technology (the internet, smartphone, social media etc.) to find health services?

| | |
|---|---|
| 56. Did you feel like the service knew how to help you? * <br>(Please tick one)<br>Please comment: | ☐ Yes<br>☐ No<br>☐ Not sure |
| **57.** Would you recommend the service you visited to other young people? * <br>(Please tick one)<br>Please comment: | ☐ Yes<br>☐ No<br>☐ Not sure |

| | |
|---|---|
| 58. If you went to multiple health services in the past 6 months, did you go for health problems that were: (Please tick one) * | ☐ Related/similar health problems<br>☐ Unrelated/different health problems<br>☐ I did not go to multiple health services |

59. How would you describe your experience in finding your way around health care services? (e.g. confusing, straightforward, took too long, frustrating, easy)

60. Would you like to share any of these experiences? If so, please tell us a story about your experience.

61. What would make it easier to find your way around health care services?

62. Do you have any suggestions about how health services can be better for young people?

## PART 7: THIS SECTION ASKS ABOUT YOUR HEALTH

| | |
|---|---|
| 63. In general would you say that your health is: * (Please tick one) | ☐ Excellent<br>☐ Very good<br>☐ Good<br>☐ Fair<br>☐ Poor |
| 64. Do you have any of the following: * (Please tick all that apply) | ☐ Chronic health condition since birth/early childhood (please specify):<br>☐ Diabetes (please specify):<br>☐ Depression, anxiety or other mental illness<br>☐ Developmental or Intellectual disability<br>☐ Drug and alcohol problems<br>☐ An eating disorder (please specify):<br>☐ Physical disability<br>☐ Overweight/obesity<br>☐ None of the above<br>☐ Other (please specify): |
| 65. **In the last month,** have you stayed away from school or work because you were sick or hurt yourself? *(Please tick one)<br>Please comment: | ☐ Yes<br>☐ No |
| 66. **In the last month,** have you stayed away from school or work to look after someone else? * (Please tick one)<br>Please comment: | ☐ Yes<br>☐ No |

67. **Anxiety and depression checklist (K10)**
This simple checklist aims to measure if you might have been affected by depression and anxiety in **the past four weeks.** Tick a box next to each question that best represents how you have been. *

| | None of the time | A little of the time | Some of the time | Most of the time | All of the time |
|---|---|---|---|---|---|
| **About how often did you feel tired out for no good reason?** | | | | | |
| **About how often did you feel nervous?** | | | | | |
| **About how often did you feel so nervous that nothing could calm you down?** | | | | | |
| **About how often did you feel hopeless?** | | | | | |
| **About how often did you feel restless or fidgety?** | | | | | |
| **About how often did you feel so restless you could not sit still?** | | | | | |
| **About how often did you feel depressed?** | | | | | |
| **About how often did you feel that everything was an effort?** | | | | | |
| **About how often did you feel so sad that nothing could cheer you up?** | | | | | |
| **About how often did you feel worthless?** | | | | | |

68. **Please indicate for each of the five statements, which is closest to how you have been feeling over the last two weeks.** *

| | All of the time | Most of the time | More than half of the time | Less than half of the time | At no time |
|---|---|---|---|---|---|
| **I have felt cheerful and in good spirits** | | | | | |
| **I have felt calm and relaxed** | | | | | |
| **I have felt active and vigorous** | | | | | |
| **I woke up feeling fresh and rested** | | | | | |
| **My daily life has been filled with things that interest me** | | | | | |

69. **Anything else?**
Please tell us anything else you would like to about access to health services and/ or online health services:

70. **Did a parent or carer or someone from school/ IEC help you to complete this survey?** *
□ **Yes** □ **No**

◊Kang M, Robards F, Sanci L, Steinbeck K, Jan S, Hawke C, Luscombe G, Kong M, Usherwood T. (2018). *Access 3*: young people and the health system in the digital age - final research report. Department of General Practice Westmead, The University of Sydney and the Australian Centre for Public and Population Health Research, The University of Technology Sydney, Australia.
